# (*E*)-*N*′-(2,4,6-Trihydroxy­benzyl­idene)isonicotinohydrazide sesquihydrate

**DOI:** 10.1107/S1600536810014959

**Published:** 2010-04-28

**Authors:** H. S. Naveenkumar, Amirin Sadikun, Pazilah Ibrahim, Wan-Sin Loh, Hoong-Kun Fun

**Affiliations:** a School of Pharmaceutical Sciences, Universiti Sains Malaysia, 11800 USM, Penang, Malaysia; b X-ray Crystallography Unit, School of Physics, Universiti Sains Malaysia, 11800 USM, Penang, Malaysia

## Abstract

In the title compound, C_13_H_11_N_3_O_4_·1.5H_2_O, the pyridine ring forms a dihedral angle of 1.50 (6)° with the benzene ring. An intra­molecular O—H⋯N hydrogen bond forms a six-membered ring with an *S*(6) ring motif. In the crystal structure, one water mol­ecule is disordered over two positions around an inversion centre with site-occupancy factors of 0.5. Inter­molecular O—H⋯N, O—H⋯O, N—H⋯O and C—H⋯O hydrogen bonds consolidate the structure into a three dimensional network. A π–π stacking inter­action with a centroid–centroid distance of 3.5949 (7) Å is also present.

## Related literature

For biological applications of isoniazid derivatives, see: Janin (2007 ▶); Maccari *et al.* (2005 ▶); Slayden & Barry (2000 ▶). For the biological activity of Schiff bases, see: Kahwa *et al.* (1986 ▶). For related structures, see: Naveenkumar *et al.* (2009 ▶); Naveenkumar, Sadikun, Ibrahim, Quah & Fun (2010 ▶); Naveenkumar, Sadikun, Ibrahim, Yeap & Fun (2010 ▶); Shi (2005 ▶). For hydrogen-bond motifs, see: Bernstein *et al.* (1995 ▶). For bond-length data, see: Allen *et al.* (1987 ▶). For the synthesis, see: Lourenco *et al.* (2008 ▶). For the stability of the temperature controller used for the data collection, see: Cosier & Glazer (1986 ▶).
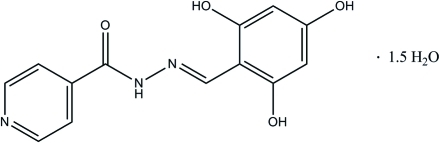


## Experimental

### 

#### Crystal data

C_13_H_11_N_3_O_4_·1.5H_2_O
*M* *_r_* = 300.27
Monoclinic, 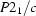

*a* = 8.4639 (1) Å
*b* = 13.2279 (2) Å
*c* = 13.4363 (2) Å
β = 120.037 (1)°
*V* = 1302.30 (3) Å^3^
*Z* = 4
Mo *K*α radiation
μ = 0.12 mm^−1^
*T* = 100 K
0.48 × 0.46 × 0.19 mm

#### Data collection

Bruker SMART APEXII CCD area-detector diffractometer
Absorption correction: multi-scan (*SADABS*; Bruker, 2009 ▶) *T*_min_ = 0.944, *T*_max_ = 0.977
14912 measured reflections
3795 independent reflections
3090 reflections with *I* > 2σ(*I*)
*R*_int_ = 0.025

#### Refinement

*R*[*F* ^2^ > 2σ(*F* ^2^)] = 0.045
*wR*(*F* ^2^) = 0.139
*S* = 1.05
3795 reflections
244 parameters
H atoms treated by a mixture of independent and constrained refinement
Δρ_max_ = 0.38 e Å^−3^
Δρ_min_ = −0.38 e Å^−3^

### 

Data collection: *APEX2* (Bruker, 2009 ▶); cell refinement: *SAINT* (Bruker, 2009 ▶); data reduction: *SAINT*; program(s) used to solve structure: *SHELXTL* (Sheldrick, 2008 ▶); program(s) used to refine structure: *SHELXTL*; molecular graphics: *SHELXTL*; software used to prepare material for publication: *SHELXTL* and *PLATON* (Spek, 2009 ▶).

## Supplementary Material

Crystal structure: contains datablocks global, I. DOI: 10.1107/S1600536810014959/is2532sup1.cif

Structure factors: contains datablocks I. DOI: 10.1107/S1600536810014959/is2532Isup2.hkl

Additional supplementary materials:  crystallographic information; 3D view; checkCIF report

## Figures and Tables

**Table 1:** Hydrogen-bond geometry (Å, °)

*D*—H⋯*A*	*D*—H	H⋯*A*	*D*⋯*A*	*D*—H⋯*A*
O1*W*—H1*W*1⋯O4	0.76	2.05	2.8134 (13)	176
O1*W*—H2*W*1⋯O2^i^	0.82	2.09	2.8886 (14)	165
O2*W*—H1*W*2⋯O4^ii^	0.83	2.06	2.864 (3)	162
O2*W*—H2*W*2⋯O4	0.83	2.17	2.844 (3)	139
N2—H1*N*2⋯O1*W*^iii^	0.87 (2)	1.99 (2)	2.8548 (13)	170 (3)
O1—H1*O*1⋯N1	0.87 (3)	1.78 (2)	2.5696 (15)	149 (2)
O2—H1*O*2⋯N3^iv^	0.87 (3)	1.82 (3)	2.6470 (14)	158 (3)
O3—H1*O*3⋯O1^v^	0.72 (3)	2.16 (3)	2.7579 (15)	142 (3)
O3—H1*O*3⋯O2*W*^vi^	0.72 (3)	2.40 (3)	2.970 (2)	138 (3)
C4—H4*A*⋯O2*W*^vi^	0.984 (18)	2.290 (17)	3.135 (2)	143.3 (14)
C7—H7*A*⋯O1*W*^iii^	0.993 (19)	2.539 (19)	3.3185 (16)	135.2 (14)
C10—H10*A*⋯O1*W*^iii^	0.996 (18)	2.355 (18)	3.3063 (17)	159.4 (13)

Symmetry codes: (i) 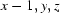; (ii) 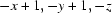; (iii) 
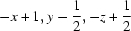
; (iv) 
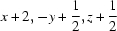
; (v) 
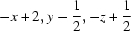
; (vi) 
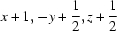
.

**Fig. 1.**
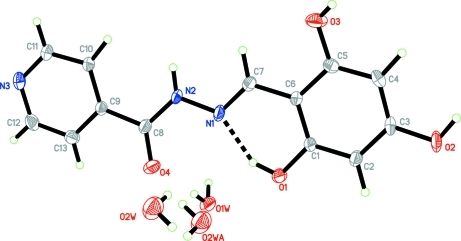
The molecular structure of the title compound, showing 50% probability displacement ellipsoids and the atom-numbering scheme. Dashed line indicates the intramolecular hydrogen bond. Atom O2WA was generated by symmetry code -x+1, -y+1, -z.

**Fig. 2.**
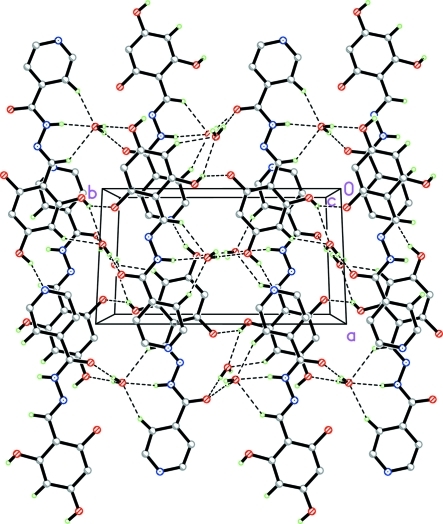
The crystal packing of the title compound, viewed along the c axis. Intermolecular interactions are shown as dashed lines. H atoms not involved in the intermolecular interactions (dashed lines) have been omitted for clarity.

## supplementary crystallographic information

### Comment

In the search of new compounds, isoniazid derivatives have been found to possess potential tuberculostatic activity (Janin, 2007; Maccari *et al.*, 2005; Slayden & Barry, 2000). Schiff bases have attracted much attention because of their biological activity (Kahwa *et al.*, 1986). As a part of a current work of synthesis of (*E*)-*N'*-(substituted-benzylidene)isonicotinohydrazide derivatives, in this paper we present the crystal structure of the title compound.

The asymmetric unit of the title compound (Fig. 1), contains one (*E*)-*N'*-(2,4,6-trihydroxybenzylidene) isonicotinohydrazide and one and a half water molecules. The partially-occupied water molecule (O2W, H1W2, H2W2) is disordered across a crystallographic inversion center. The pyridine ring (C9–C11/N3/C12/C13) is essentially planar with a maximum deviation of 0.006 (1) Å at atom C9 and forms a dihedral angle of 1.51 (6)° with the benzene ring (C1–C6). An intramolecular O1—H1O1···N1 hydrogen bond forms a six-membered ring with an *S*(6) ring motif (Bernstein *et al.*, 1995). The bond lengths are within normal values (Allen *et al.*, 1987) and are comparable to those observed for closely related structures (Naveenkumar *et al.*,  2009; Naveenkumar, Sadikun, Ibrahim, Quah & Fun, 2010; Naveenkumar, Sadikun, Ibrahim, Yeap & Fun, 2010; Shi, 2005).

In the crystal packing (Fig. 2), intermolecular O1W—H1W1···O4, O1W—H2W1···O2, O2W—H1W2···O4, O2W—H2W2···O4, N2—H1N2···O1W, O2—H1O2···N3, O3—H1O3···O1, O3—H1O3···O2W, C4—H4A···O2W, C7—H7A···O1W and C10—H10A···O1W hydrogen bonds (Table 1) consolidate the structure into a three dimensional network. The crystal structure is further stabilized by π–π stacking interactions involving the pyridine (*Cg*1) and benzene (*Cg*2) rings with a centroid–centroid distance of 3.5949 (7) Å (symmetry code = -1+*x*, *y*, *z*).

### Experimental

The isoniazid derivative was prepared following the procedure by Lourenco *et al.* (2008). (*E*)-*N'*-(2,4,6-trihydroxybenzylidene)isonicotinohydrazide hydrate was prepared by reaction between the 2,4,6-trihydroxy benzaldehyde (1.0 eq) with isoniazid (1.0 eq) in ethanol/water. After stirring for 1 to 3 h at room temperature, the resulting mixture was concentrated under reduced pressure. The residue after being purified by washing with cold ethanol and ethyl ether, afforded the pure derivative. Colourless single crystals suitable for X-ray analysis were obtained by recrystallization with methanol.

### Refinement

All the H atoms were located from a difference Fourier map. H1W1, H2W1, H1W2 and H2W2 were allowed to ride on their parent atoms to which they were attached, with *U*_iso_(H) = 1.5*U*_eq_(parent atom). The remaining H were refined freely. [O—H = 0.74 (3)–0.974 (10) Å, N—H = 0.88 (2) Å and  C—H = 0.895 (19)–1.025 (18) Å]. The partially-occupied disordered water molecule was fixed at 50% occupancy in the final refinement.

### Crystal data

**Table:**

C_13_H_11_N_3_O_4_·1.5H_2_O	*F*(000) = 628
*M**_r_* = 300.27	*D*_x_ = 1.531 Mg m^−^^3^
Monoclinic, *P*2_1_/*c*	Mo *K*α radiation, λ = 0.71073 Å
Hall symbol: -P 2ybc	Cell parameters from 6535 reflections
*a* = 8.4639 (1) Å	θ = 2.3–30.0°
*b* = 13.2279 (2) Å	µ = 0.12 mm^−^^1^
*c* = 13.4363 (2) Å	*T* = 100 K
β = 120.037 (1)°	Block, brown
*V* = 1302.30 (3) Å^3^	0.48 × 0.46 × 0.19 mm
*Z* = 4	

### Data collection

**Table:**

Bruker SMART APEXII CCD area-detector diffractometer	3795 independent reflections
Radiation source: fine-focus sealed tube	3090 reflections with *I* > 2σ(*I*)
graphite	*R*_int_ = 0.025
φ and ω scans	θ_max_ = 30.0°, θ_min_ = 2.3°
Absorption correction: multi-scan (*SADABS*; Bruker, 2009)	*h* = −11→9
*T*_min_ = 0.944, *T*_max_ = 0.977	*k* = −16→18
14912 measured reflections	*l* = −18→18

### Refinement

**Table:**

Refinement on *F*^2^	Primary atom site location: structure-invariant direct methods
Least-squares matrix: full	Secondary atom site location: difference Fourier map
*R*[*F*^2^ > 2σ(*F*^2^)] = 0.045	Hydrogen site location: inferred from neighbouring sites
*wR*(*F*^2^) = 0.139	H atoms treated by a mixture of independent and constrained refinement
*S* = 1.05	*w* = 1/[σ^2^(*F*_o_^2^) + (0.086*P*)^2^ + 0.1988*P*] where *P* = (*F*_o_^2^ + 2*F*_c_^2^)/3
3795 reflections	(Δ/σ)_max_ = 0.001
244 parameters	Δρ_max_ = 0.38 e Å^−^^3^
0 restraints	Δρ_min_ = −0.38 e Å^−^^3^

### Special details

**Table:**

Experimental. The crystal was placed in the cold stream of an Oxford Cryosystems Cobra open-flow nitrogen cryostat (Cosier & Glazer, 1986) operating at 100.0 (1) K.
Geometry. All esds (except the esd in the dihedral angle between two l.s. planes) are estimated using the full covariance matrix. The cell esds are taken into account individually in the estimation of esds in distances, angles and torsion angles; correlations between esds in cell parameters are only used when they are defined by crystal symmetry. An approximate (isotropic) treatment of cell esds is used for estimating esds involving l.s. planes.
Refinement. Refinement of *F*^2^ against ALL reflections. The weighted *R*-factor wR and goodness of fit *S* are based on *F*^2^, conventional *R*-factors *R* are based on *F*, with *F* set to zero for negative *F*^2^. The threshold expression of *F*^2^ > σ(*F*^2^) is used only for calculating *R*-factors(gt) etc. and is not relevant to the choice of reflections for refinement. *R*-factors based on *F*^2^ are statistically about twice as large as those based on *F*, and *R*- factors based on ALL data will be even larger.

### Fractional atomic coordinates and isotropic or equivalent isotropic displacement parameters (Å^2^)

**Table:**

	*x*	*y*	*z*	*U*_iso_*/*U*_eq_	Occ. (<1)
O1W	0.52172 (13)	0.55973 (6)	0.31668 (9)	0.0306 (2)	
H1W1	0.4784	0.5243	0.2649	0.046*	
H2W1	0.5174	0.5182	0.3604	0.046*	
O2W	0.4201 (3)	0.47492 (16)	−0.05523 (19)	0.0394 (5)	0.50
H1W2	0.4991	0.5075	−0.0609	0.059*	0.50
H2W2	0.4565	0.4623	0.0132	0.059*	0.50
O2	1.48904 (12)	0.38723 (8)	0.43699 (8)	0.0293 (2)	
O3	1.05057 (15)	0.11707 (7)	0.32046 (9)	0.0306 (2)	
O4	0.34364 (13)	0.43232 (6)	0.12386 (8)	0.0280 (2)	
C1	0.99950 (15)	0.38970 (8)	0.31225 (10)	0.0201 (2)	
C2	1.17860 (16)	0.42239 (9)	0.35826 (11)	0.0232 (3)	
C3	1.31689 (15)	0.35102 (9)	0.39166 (10)	0.0209 (2)	
C4	1.27822 (16)	0.24762 (9)	0.37910 (10)	0.0216 (2)	
C5	1.09857 (16)	0.21590 (8)	0.33268 (9)	0.0193 (2)	
C6	0.95461 (15)	0.28593 (8)	0.29762 (9)	0.0169 (2)	
C7	0.76941 (16)	0.25025 (8)	0.25037 (9)	0.0188 (2)	
C8	0.32356 (16)	0.34043 (8)	0.13119 (10)	0.0192 (2)	
C9	0.13728 (15)	0.29709 (8)	0.08915 (9)	0.0168 (2)	
C10	0.09871 (15)	0.19404 (8)	0.07857 (10)	0.0185 (2)	
C11	−0.08123 (16)	0.16375 (9)	0.03428 (10)	0.0204 (2)	
C12	−0.18124 (17)	0.32760 (10)	0.01401 (11)	0.0247 (3)	
C13	−0.00643 (17)	0.36473 (9)	0.05683 (11)	0.0229 (2)	
N1	0.63769 (13)	0.31447 (7)	0.21721 (8)	0.0204 (2)	
N2	0.46426 (13)	0.27539 (8)	0.17637 (8)	0.0190 (2)	
N3	−0.22009 (14)	0.22835 (8)	0.00221 (8)	0.0223 (2)	
O1	0.86777 (13)	0.46088 (7)	0.28226 (10)	0.0343 (3)	
H2A	1.208 (2)	0.4987 (14)	0.3659 (15)	0.040 (4)*	
H4A	1.371 (3)	0.1941 (13)	0.4038 (14)	0.034 (4)*	
H7A	0.751 (2)	0.1759 (14)	0.2465 (14)	0.038 (5)*	
H10A	0.191 (2)	0.1392 (12)	0.1008 (14)	0.030 (4)*	
H11A	−0.111 (2)	0.0914 (12)	0.0240 (14)	0.031 (4)*	
H12A	−0.275 (3)	0.3696 (13)	−0.0081 (15)	0.038 (4)*	
H13A	0.015 (3)	0.4324 (14)	0.0657 (15)	0.040 (5)*	
H1N2	0.456 (3)	0.2097 (15)	0.1781 (16)	0.042 (5)*	
H1O1	0.763 (3)	0.4296 (17)	0.2534 (18)	0.067 (7)*	
H1O2	1.567 (4)	0.3379 (19)	0.460 (2)	0.082 (8)*	
H1O3	1.115 (4)	0.083 (2)	0.318 (2)	0.091 (9)*	

### Atomic displacement parameters (Å^2^)

**Table:**

	*U*^11^	*U*^22^	*U*^33^	*U*^12^	*U*^13^	*U*^23^
O1W	0.0255 (5)	0.0203 (4)	0.0424 (6)	−0.0037 (3)	0.0141 (4)	−0.0053 (4)
O2W	0.0331 (11)	0.0438 (12)	0.0422 (12)	−0.0075 (9)	0.0195 (10)	−0.0002 (9)
O2	0.0091 (4)	0.0362 (5)	0.0367 (5)	−0.0001 (3)	0.0070 (4)	−0.0033 (4)
O3	0.0389 (6)	0.0164 (4)	0.0446 (6)	0.0028 (4)	0.0270 (5)	−0.0006 (4)
O4	0.0243 (5)	0.0181 (4)	0.0412 (5)	−0.0043 (3)	0.0159 (4)	0.0009 (3)
C1	0.0129 (5)	0.0171 (5)	0.0280 (6)	0.0008 (4)	0.0084 (5)	−0.0002 (4)
C2	0.0152 (5)	0.0197 (5)	0.0339 (6)	−0.0024 (4)	0.0116 (5)	−0.0053 (4)
C3	0.0104 (5)	0.0295 (6)	0.0202 (5)	−0.0005 (4)	0.0056 (4)	−0.0026 (4)
C4	0.0172 (6)	0.0261 (6)	0.0211 (5)	0.0076 (4)	0.0092 (5)	0.0031 (4)
C5	0.0219 (6)	0.0181 (5)	0.0193 (5)	0.0026 (4)	0.0114 (5)	0.0009 (4)
C6	0.0137 (5)	0.0174 (5)	0.0169 (5)	−0.0011 (4)	0.0057 (4)	−0.0002 (4)
C7	0.0178 (5)	0.0200 (5)	0.0170 (5)	−0.0042 (4)	0.0076 (4)	−0.0005 (4)
C8	0.0161 (5)	0.0204 (5)	0.0195 (5)	−0.0037 (4)	0.0078 (4)	−0.0004 (4)
C9	0.0139 (5)	0.0194 (5)	0.0163 (5)	−0.0016 (4)	0.0068 (4)	0.0007 (4)
C10	0.0132 (5)	0.0200 (5)	0.0201 (5)	−0.0012 (4)	0.0067 (4)	0.0003 (4)
C11	0.0146 (5)	0.0242 (6)	0.0206 (5)	−0.0041 (4)	0.0073 (4)	−0.0024 (4)
C12	0.0169 (6)	0.0289 (6)	0.0277 (6)	0.0051 (5)	0.0109 (5)	0.0052 (5)
C13	0.0209 (6)	0.0202 (5)	0.0278 (6)	0.0017 (4)	0.0124 (5)	0.0035 (4)
N1	0.0117 (4)	0.0254 (5)	0.0200 (5)	−0.0053 (4)	0.0048 (4)	0.0015 (4)
N2	0.0114 (4)	0.0199 (5)	0.0215 (5)	−0.0052 (3)	0.0052 (4)	0.0005 (3)
N3	0.0135 (4)	0.0324 (5)	0.0195 (5)	−0.0008 (4)	0.0073 (4)	0.0004 (4)
O1	0.0166 (5)	0.0175 (4)	0.0695 (7)	0.0026 (3)	0.0221 (5)	0.0055 (4)

### Geometric parameters (Å, °)

**Table:**

O1W—H1W1	0.7630	C6—C7	1.4445 (15)
O1W—H2W1	0.8193	C7—N1	1.2908 (15)
O2W—O2W^i^	1.569 (4)	C7—H7A	0.994 (18)
O2W—H1W2	0.8308	C8—N2	1.3428 (15)
O2W—H2W2	0.8278	C8—C9	1.4973 (15)
O2—C3	1.3546 (14)	C9—C10	1.3923 (15)
O2—H1O2	0.87 (3)	C9—C13	1.3928 (16)
O3—C5	1.3544 (14)	C10—C11	1.3881 (15)
O3—H1O3	0.72 (3)	C10—H10A	0.998 (17)
O4—C8	1.2380 (13)	C11—N3	1.3380 (15)
C1—O1	1.3573 (14)	C11—H11A	0.981 (16)
C1—C2	1.3888 (16)	C12—N3	1.3434 (16)
C1—C6	1.4115 (15)	C12—C13	1.3817 (17)
C2—C3	1.3918 (16)	C12—H12A	0.892 (19)
C2—H2A	1.033 (18)	C13—H13A	0.908 (18)
C3—C4	1.3968 (17)	N1—N2	1.3845 (13)
C4—C5	1.3884 (17)	N2—H1N2	0.87 (2)
C4—H4A	0.984 (18)	O1—H1O1	0.87 (3)
C5—C6	1.4107 (15)		
			
H1W1—O1W—H2W1	93.9	C6—C7—H7A	117.1 (10)
O2W^i^—O2W—H1W2	61.0	O4—C8—N2	122.62 (11)
O2W^i^—O2W—H2W2	50.9	O4—C8—C9	120.43 (10)
H1W2—O2W—H2W2	109.7	N2—C8—C9	116.95 (9)
C3—O2—H1O2	110.3 (18)	C10—C9—C13	118.27 (11)
C5—O3—H1O3	115 (2)	C10—C9—C8	124.21 (10)
O1—C1—C2	117.87 (10)	C13—C9—C8	117.51 (10)
O1—C1—C6	120.62 (10)	C11—C10—C9	118.50 (11)
C2—C1—C6	121.50 (10)	C11—C10—H10A	116.6 (9)
C1—C2—C3	119.12 (10)	C9—C10—H10A	124.9 (9)
C1—C2—H2A	120.4 (10)	N3—C11—C10	123.51 (11)
C3—C2—H2A	120.4 (10)	N3—C11—H11A	117.2 (10)
O2—C3—C2	116.54 (11)	C10—C11—H11A	119.3 (10)
O2—C3—C4	122.33 (11)	N3—C12—C13	122.98 (11)
C2—C3—C4	121.13 (11)	N3—C12—H12A	116.4 (11)
C5—C4—C3	119.20 (10)	C13—C12—H12A	120.7 (11)
C5—C4—H4A	116.4 (10)	C12—C13—C9	119.18 (11)
C3—C4—H4A	124.4 (10)	C12—C13—H13A	120.3 (12)
O3—C5—C4	122.73 (11)	C9—C13—H13A	120.5 (12)
O3—C5—C6	115.90 (10)	C7—N1—N2	116.89 (10)
C4—C5—C6	121.35 (10)	C8—N2—N1	117.66 (10)
C5—C6—C1	117.70 (10)	C8—N2—H1N2	126.0 (13)
C5—C6—C7	119.87 (10)	N1—N2—H1N2	116.2 (13)
C1—C6—C7	122.43 (10)	C11—N3—C12	117.55 (10)
N1—C7—C6	119.76 (10)	C1—O1—H1O1	107.7 (15)
N1—C7—H7A	123.1 (10)		
			
O1—C1—C2—C3	178.88 (11)	C1—C6—C7—N1	1.73 (16)
C6—C1—C2—C3	−0.63 (19)	O4—C8—C9—C10	170.29 (11)
C1—C2—C3—O2	−179.37 (10)	N2—C8—C9—C10	−9.79 (16)
C1—C2—C3—C4	0.32 (19)	O4—C8—C9—C13	−8.54 (16)
O2—C3—C4—C5	179.54 (10)	N2—C8—C9—C13	171.38 (10)
C2—C3—C4—C5	−0.15 (18)	C13—C9—C10—C11	1.26 (16)
C3—C4—C5—O3	−178.26 (10)	C8—C9—C10—C11	−177.57 (10)
C3—C4—C5—C6	0.26 (17)	C9—C10—C11—N3	−0.76 (17)
O3—C5—C6—C1	178.08 (10)	N3—C12—C13—C9	0.27 (19)
C4—C5—C6—C1	−0.54 (16)	C10—C9—C13—C12	−1.03 (17)
O3—C5—C6—C7	−0.98 (15)	C8—C9—C13—C12	177.87 (10)
C4—C5—C6—C7	−179.60 (10)	C6—C7—N1—N2	−177.87 (9)
O1—C1—C6—C5	−178.77 (10)	O4—C8—N2—N1	0.86 (17)
C2—C1—C6—C5	0.73 (17)	C9—C8—N2—N1	−179.06 (9)
O1—C1—C6—C7	0.27 (17)	C7—N1—N2—C8	−173.68 (10)
C2—C1—C6—C7	179.76 (11)	C10—C11—N3—C12	−0.01 (17)
C5—C6—C7—N1	−179.26 (10)	C13—C12—N3—C11	0.26 (18)

Symmetry codes: (i) −*x*+1, −*y*+1, −*z*.

### Hydrogen-bond geometry (Å, °)

**Table:**

*D*—H···*A*	*D*—H	H···*A*	*D*···*A*	*D*—H···*A*
O1W—H1W1···O4	0.76	2.05	2.8134 (13)	176
O1W—H2W1···O2^ii^	0.82	2.09	2.8886 (14)	165
O2W—H1W2···O4^i^	0.83	2.06	2.864 (3)	162
O2W—H2W2···O4	0.83	2.17	2.844 (3)	139
N2—H1N2···O1W^iii^	0.87 (2)	1.99 (2)	2.8548 (13)	170 (3)
O1—H1O1···N1	0.87 (3)	1.78 (2)	2.5696 (15)	149 (2)
O2—H1O2···N3^iv^	0.87 (3)	1.82 (3)	2.6470 (14)	158 (3)
O3—H1O3···O1^v^	0.72 (3)	2.16 (3)	2.7579 (15)	142 (3)
O3—H1O3···O2W^vi^	0.72 (3)	2.40 (3)	2.970 (2)	138 (3)
C4—H4A···O2W^vi^	0.984 (18)	2.290 (17)	3.135 (2)	143.3 (14)
C7—H7A···O1W^iii^	0.993 (19)	2.539 (19)	3.3185 (16)	135.2 (14)
C10—H10A···O1W^iii^	0.996 (18)	2.355 (18)	3.3063 (17)	159.4 (13)

Symmetry codes: (ii) *x*−1, *y*, *z*; (i) −*x*+1, −*y*+1, −*z*; (iii) −*x*+1, *y*−1/2, −*z*+1/2; (iv) *x*+2, −*y*+1/2, *z*+1/2; (v) −*x*+2, *y*−1/2, −*z*+1/2; (vi) *x*+1, −*y*+1/2, *z*+1/2.
